# Supplementary material for: The Primary Causes of Muscle Dysfunction Associated with the Point Mutations in Tpm3.12; Conformational Analysis of Mutant Proteins as a Tool for Classification of Myopathies
Source: Int J Mol Sci. 2018 Dec 10;19(12):3975. doi: 10.3390/ijms19123975 (PMC6321504; doi:10.3390/ijms19123975)
Supplement: Supplementary file 1 [file ijms-19-03975-s001.pdf]

Supplementary materials 1: Mathematical fitting of the parameters

The Φ_A, Φ_E, N, and θ are chosen by mathematical analysis to give the best fit to the observed values of the ratios $_{\perp}I_{\perp}/_{||}I_{||}$, $_{||}I_{\perp}/_{||}I_{||}$, and $_{\perp}I_{||}/_{||}I_{||}$ (Yanagida and Oosawa, 1978; Kakol *et al.*, 1987; Borovikov *et al.*, 2004) with use of following equations:

$$_{||}I_{||} = (\sin^2\Theta_{1/2} - \sin^4\Theta_{1/2}) \times ((1/2) \times \sin^2\Phi_A \times \cos^2\Phi_E - \cos^2\Phi_A \times \cos^2\Phi_E$$
$$+ (1/2) \times \sin^2\Phi_E \times \cos^2\Phi_A + 2 \times \cos\delta \times \sin\Phi_A \times \sin\Phi_E \times \cos\Phi_A \times \cos\Phi_E)$$
$$+ (1 - \sin^2\Theta_{1/2}) \times (1 - \sin^2\Phi_A - \sin^2\Phi_E)$$
$$+ ((1 - \sin^2\Theta_{1/2} + (1/8) \times (1 + \cos^2\delta) \times \sin^4\Theta_{1/2}) \times \sin^2\Phi_A \times \sin^2\Phi_E ;$$

$$_{||}I_{\perp} = (\sin^2\Theta_{1/2} - \sin^4\Theta_{1/2}) \times ((1/2) \times \cos^2\Phi_A \times \cos^2\Phi_E - (1/4) \times \sin^2\Phi_E \times \cos^2\Phi_A$$
$$- (1/4) \times \sin^2\Phi_A \times \cos^2\Phi_E - \cos\delta \times \sin\Phi_A \times \sin\Phi_E \times \cos\Phi_A \times \cos\Phi_E)$$
$$+ (1/2) \times (1 - \sin^2\Theta_{1/2}) \times (1 - \sin^2\Phi_A - \cos^2\Phi_E)$$
$$+ (1/2) \times (1 - (1/2) \times \sin^2\Theta_{1/2}) \times \sin^2\Phi_A \times \cos^2\Phi_E$$
$$+ (1/8) \times (2 \times \sin^2\Theta_{1/2} - ((1/2)+ \cos^2\delta) \times \sin^4\Theta_{1/2}) \times \sin^2\Phi_A \times \sin^2\Phi_E ;$$

$$_{\perp}I_{||} = (\sin^2\Theta_{1/2} - \sin^4\Theta_{1/2}) \times ((1/2) \times \cos^2\Phi_E \times \cos^2\Phi_A - (1/4) \times \sin^2\Phi_A \times \cos^2\Phi_E$$
$$- (1/4) \times \sin^2\Phi_E \times \cos^2\Phi_A - \cos\delta \times \sin\Phi_A \times \sin\Phi_E \times \cos\Phi_A \times \cos\Phi_E)$$
$$+ (1/2) \times (1 - \sin^2\Theta_{1/2}) \times (1 - \sin^2\Phi_E - \cos^2\Phi_A)$$
$$+ (1/2) \times (1 - (1/2) \times \sin^2\Theta_{1/2}) \times \sin^2\Phi_E \times \cos^2\Phi_A$$
$$+ (1/8) \times (2 \times \sin^2\Theta_{1/2} - ((1/2) + \cos^2\delta) \times \sin^4\Theta_{1/2}) \times \sin^2\Phi_E \times \sin^2\Phi_A ;$$

$$_{\perp}I_{\perp} = (\sin^2\Theta_{1/2} - (3/4) \times \sin^4\Theta_{1/2}) \times ((1/4) \times \sin^2\Phi_A \times \cos^2\Phi_E + (1/4) \times \sin^2\Phi_E \times \cos^2\Phi_A$$
$$- (1/16) \times (1 + 2 \times \cos^2\delta) \times \sin^2\Phi_A \times \sin^2\Phi_E + \cos\delta \times \sin\Phi_A \times \sin\Phi_E \times \cos\Phi_A \times \cos\Phi_E)$$
$$+ (1/16) \times (2 - \sin^2\Theta_{1/2}) \times (1 + 2 \times \cos^2\delta) \times (1 - \cos^2\Phi_A - \cos^2\Phi_E)$$
$$+ (1/8) \times (3 \times \sin^4\Theta_{1/2} - ((1/2) \times \sin^2\Theta_{1/2} - 1) \times (1 + 2 \times \cos^2\delta) \times \cos^2\Phi_A \times \cos^2\Phi_E ;$$
$$\cos\gamma = \cos^2\Phi_A \times \cos^2\Phi_E + \sin\Phi_A \times \sin\Phi_E \times \cos^2\delta ;$$

$$\frac{_{//}I_{\perp}}{_{//}I_{//}} = \frac{(15/4)\times(1-N)\times\cos^2\Phi_A\times\sin^2\Phi_E+2\times N\times(2-\cos^2\gamma)}{30\times(1-N)\times\cos^2\Phi_A\times\cos^2\Phi_E+2\times N\times(1+2\cos^2\gamma)} \quad .$$
